# Safety and Immunogenicity of a *Mycoplasma ovipneumoniae* Bacterin for Domestic Sheep (*Ovis aries*)

**DOI:** 10.1371/journal.pone.0095698

**Published:** 2014-04-21

**Authors:** Jessie C. Ziegler, Kevin K. Lahmers, George M. Barrington, Steven M. Parish, Katherine Kilzer, Katherine Baker, Thomas E. Besser

**Affiliations:** 1 Department of Veterinary Clinical Medicine, Washington State University, Pullman, Washington, United States of America; 2 Department of Veterinary Microbiology and Pathology, Washington State University, Pullman, Washington, United States of America; The Ohio State University, United States of America

## Abstract

**Background:**

Mortality from epizootic pneumonia is hindering re-establishment of bighorn sheep populations in western North America. *Mycoplasma ovipneumoniae*, a primary agent of this disease, is frequently carried asymptomatically by the domestic sheep and goats that constitute the reservoir of this agent for transmission to bighorn sheep. Our long-term objective is to reduce the risk of *M. ovipneumoniae* infection of bighorn sheep; one approach to this objective is to control the pathogen in its reservoir hosts.

**Methods:**

The safety and immunogenicity of *M. ovipneumoniae* for domestic sheep was evaluated in three experimental immunization protocols: 1) live *M. ovipneumoniae* (50 ug protein); 2) killed *M. ovipneumoniae* (50 ug whole cell protein) in oil adjuvant; and 3) killed *M. ovipneumoniae* (250 ug whole cell protein) in oil adjuvant. Immunogenicity was assessed by two serum antibody measures: competitive enzyme-linked immunosorbent assay (cELISA) (experiments 1–3) and serum growth inhibition (Experiment 3). Passive immunogenicity was also assessed in the third experiment using the same assays applied to blood samples obtained from the lambs of immunized ewes.

**Results and Conclusions:**

Adverse reactions to immunization were generally minor, but local reactions were regularly observed at immunization sites with bacterins in oil adjuvants. No evidence of *M. ovipneumoniae* specific antibody responses were observed in the first or second experiments and no resistance to colonization was observed in the first experiment. However, the ewes in the third experiment developed strong cELISA serum antibody responses and significant serum *M. ovipneumoniae* inhibition activity, and these responses were passively transferred to their lambs. The results of these trials indicate that immunization with relatively large antigenic mass combined with an adjuvant is capable of inducing strong active antibody responses in ewes and passively immunizing lambs.

## Introduction

Pneumonia epizootics have played a major role in the decline of bighorn sheep *(Ovis canadensis)* populations in the United States [1], [2], but the specific cause of bighorn sheep pneumonia has been debated for some time. *Mannheimia haemolytica, Bibersteinia trehalosi, Pasteurella multocida*, and *Mycoplasma ovipneumoniae* are all frequently detected in affected lung tissues [1]–[6]. Contacts between domestic sheep and goats have frequently been observed to precede bighorn sheep pneumonia outbreaks, and experimental contact with domestic sheep results in fatal pneumonia in >95% of bighorn sheep [3]–[11]. Recent evidence supports the hypothesis that *M. ovipneumoniae* is the primary agent responsible for these outbreaks but acts indirectly by impairing pulmonary defenses, predisposing to polymicrobial pneumonia with multiple secondary bacterial agents [1], [2], [6]. According to this hypothesis, *M. ovipneumoniae*, a pathogen frequently carried by domestic sheep and goats but absent from healthy bighorn sheep populations, triggers pneumonia epizootics involving animals of all ages when introduced to naïve bighorn sheep populations. Bighorn sheep that survive the all-ages epizootic become immune but some individuals continue to carry *M. ovipneumoniae* in their upper respiratory tract, serving as a source of infection to lambs. As a result, annual lamb pneumonia epizootics may recur for many years after the initial all-ages outbreak [12].

The *M. ovipneumoniae* hypothesis suggests novel avenues for control and prevention of disease in bighorn sheep [2]. Past efforts to prevent pneumonia in bighorn sheep have centered on immunization against Pasteurelleceae bacteria and their toxins, especially *M. haemolytica* and its leukotoxin. Early studies by Foreyt utilizing multivalent bacterin-toxoid vaccines for *M. haemolytica* A1, A2, and *B. trehalosi* T10 proved unsuccessful at preventing disease and death after experimental challenge [13], [14]. Foreyt also evaluated a cytotoxic A11 strain of *M. haemolytica* as a candidate live bacterial vaccine; while the A11 strain was non-lethal to bighorn sheep, it also failed to protect bighorn sheep from experimental challenge with the virulent A2 strain [15]. Cassirer et al. immunized bighorn ewes that had survived a pneumonia epizootic against *M. haemolytica* in an unsuccessful attempt to improve passive immune protection of bighorn lambs [16]. Finally, Subramaniam et al. (2011) used five doses of a multivalent *Mannheimia-Bibersteinia* vaccine to induce high titers of leukotoxin-neutralizing antibodies and antibodies against surface antigens; a protocol that protected bighorn sheep against homologous challenge [17], but the efficacy of this protocol in protecting from natural exposure remains unknown.

Even if a vaccine capable of consistently protecting bighorn sheep from *M. haemolytica* and other Pasteurellaceae is developed, obstacles remain. First, specific immunity to Pasteurellaceae may not effectively protect bighorn sheep from the polymicrobial pneumonia following *M. ovipneumoniae* infection. Second, delivery of any vaccine protocol to a wildlife species, particularly in species (like bighorn sheep) that inhabit steep and inaccessible terrain, presents considerable practical difficulties [13]–[16], [18]–[20].

Therefore, we decided to evaluate an indirect approach to prevention of bighorn sheep pneumonia by targeting the domestic small ruminant reservoirs of the pathogens. We have previously demonstrated that the risk of bighorn sheep pneumonia following contact with domestic sheep is significantly reduced in the absence of *M. ovipneumoniae* [21]. Therefore, if *M. ovipneumoniae* carriage by domestic sheep and goats adjacent to bighorn sheep habitat can be reduced or eliminated, one might expect a corresponding reduction in the risk of bighorn sheep pneumonia [2]. *M. ovipneumoniae* is ubiquitously distributed in domestic sheep and goat populations, so an effective vaccine may need to reduce carriage in currently colonized herds and flocks [22], [23]. Vaccination has been attempted in other respiratory mycoplasma diseases with varying results: control of contagious bovine pleuropneumonia (*M. mycoides* var. *mycoides*) has clearly benefited from vaccine-based approaches [24], [25], while control of atypical pneumonia of swine (*M. hyopneumoniae*) with broadly protective vaccines has not succeeded despite decades of effort [2], [26], [27].

Our longterm goal is to identify interventions that reduce or eliminate *M. ovipneumoniae* shedding by domestic sheep or goats in order to reduce or prevent disease transmission to bighorn sheep. Our specific objective in this study was to assess the safety and immunogenicity of different approaches to *M. ovipneumoniae* immunization of domestic sheep.

## Methods

### Animal use

Three immunization experiments and one challenge/colonization experiment were carried out during this study. All experiments were carried out in accordance with, and were approved by the Institutional Animal Care and Use Committee (IACUC) at Washington State University (WSU) following the recommendations in the Guide for the Care and Use of Laboratory Animals [28].

### Microbiological status of experimental animals

*M. ovipneumoniae* status was established by whole-flock testing of each flock that provided sheep for these experiments, using realtime polymerase chain reaction (RT-PCR) tests of deep nasal swab samples for *M. ovipneumoniae* shedding and competitive enzyme-linked immunosorbent assay (cELISA) tests on serum samples for *M. ovipneumoniae* specific antibodies [1]. RT-PCR and cELISA tests were conducted by the Washington Animal Disease Diagnostic Laboratory (WADDL; validation data and SOPs for the assays may be obtained directly from the laboratory at http://www.vetmed.wsu.edu/depts_waddl/). The realtime PCR assay uses primers (Movip 226F, 5′ GGGGTGCGCAACATTAGTTA 3′; LMR1, 5′-GACTTCATCCTGCACTCTGT-3′; and probe Movip 253P, 5′6-FAM-TTAGCGGGGCCAAGAGGCTGTA-BHQ-1-3′) and other reaction constituents and running conditions as described [29].

### Experimental immunization protocols

The specific experimental protocols for the three experiments are summarized in Table 1. In each experiment, animals were subcutaneously immunized twice on experimental days 1 and 28 days. Bacterin doses and adjuvants used in each experiment differed, as described in the following paragraph. In experiments 1 and 2, sham immunization with PBS lacking *M. ovipneumoniae* antigen was performed on the contralateral leg at each immunization date to serve as a control for immunization-associated inflammation. Sheep were monitored for vaccination site reactions and rectal temperatures were recorded daily for 7 days following each immunization. Serum samples for cELISA and deep nasal swab samples for *M. ovipneumoniae* RT-PCR were obtained from all animals prior to the first immunization (day 0), and then on days 7, 14, 28, 42, and 56 days subsequently. These time points were selected to evaluate the time of potential seroconversion and to correlate it with shedding status. Whole blood samples for complete blood counts and plasma fibrinogen were obtained on days 0, 3, 28, and 31 (i.e., on the day before and three days following each immunization dose) to evaluate local and systemic inflammation following vaccination. Experiment 1 included 2 ewe and 2 wether mixed breed sheep, 2 to 5 years of age, that originated from an *M. ovipneumoniae* negative flock and that tested negative for *M. ovipneumoniae* by both RT- PCR and cELISA prior to the experiment. Prior to the start of the Experiment 1, two of these sheep had previously been experimentally infected with *M. ovipneumoniae*, seroconverted, and subsequently reverted to negative status determined by repeated RT-PCR and cELISA testing. Real-time PCR and cELISA tests from the other two sheep were consistently negative. Experiment 2 included 3 ewes and 2 wethers, 3 to 7 years of age, that originated from an *M. ovipneumoniae* positive flock and all Experiment 2 sheep were RT-PCR and/or cELISA positive for *M. ovipneumoniae* at the start of the experiment. Experiment 3 included 9 pregnant ewes and 1 ram, 2 to 7 years of age, that originated from an *M. ovipneumoniae* negative flock and that tested negative for *M. ovipneumoniae* by both real-time PCR and cELISA prior to the experiment. Experiment 3 animals were randomly allocated (www.random.org/lists) to immunized and control groups of five sheep each. The ewes in Experiment 3 were pregnant at approximately 100 days gestation at the start of the experiment and lambed between days 50 and 60 of the experiment.

**Table 1:** Summary of procedures and animal sampling utilized in the immunization experiments.

	N	Vaccine Content	Vaccine Schedule (days)	cELISA/RT-PCR (days)	CBC/fibrinogen (days)	Skin Biopsies (days)
**Experiment 1**	4	50 ug cells	1, 28 d	0, 7, 14, 28, 42, 56	0, 3, 28, 31	14, 42
**Experiment 2**	5	50 ug cells+oil	1, 28 d	0, 7, 14, 28, 42, 56	0, 3, 28, 31	14, 42
**Experiment 3**						
**Adults**	10	250 ug cells+oil	1, 28 d	0, 7, 14, 28, 42, 56	0, 3, 28, 31	ND
**Lambs**	17	ND	ND	First week*	ND	ND

*Lambs birth dates were noted and blood samples were taken once for cELISA only when lambs were 1–7 days old. Lambs were not sampled past 7 days of age.

The procedures used in experiments 1–3 differed with regard to several specific protocol or sampling issues. In Experiment 1, immunizations were administered subcutaneously in the medial thigh region, but in subsequent experiments immunization were administered subcutaneously in the axilla to permit access with less animal restraint. In experiments 1 and 2, full thickness dermal punch biopsies (4 mm diameter) were obtained on experimental days 14 and 42 (i.e., two weeks following each immunization dose) in order to assess histologic evidence of inflammation and to test for *M ovipneumoniae* by RT-PCR but this was not done in experiment 3. Also in Experiment 3, blood samples for serum extraction were obtained at 24 hrs – 7 d of age from the lambs born to the experimental ewes, in order to assess their absorption of colostral antibodies, which was not possible in the non-pregnant ewes used in experiments 1 and 2.

### Bacterin preparation

*M. ovipneumoniae* strain DS11-14153, obtained from a naturally colonized domestic sheep in a WSU research flock, was broth enriched (3 d, 5% CO_2_, 37C) in Hayflick's medium [30]. Bacteria were washed three times by cycles of centrifugation (4,000×G, 15 min) and re-suspension (0.5 ml phosphate buffered saline, PBS) then stored (−20°C) until used. The protein content of bacterial suspensions was determined using the BioRad Quick Start™ Bradford Protein Assay (BioRad, Hercules, CA), and preparations were pooled, diluted and aliquoted as needed to prepare 500 ul immunization doses. Each Experiment 1 immunization dose consisted of live *M. ovipneumoniae* (50 ug protein equivalent) in PBS without adjuvant. Each immunization dose in experiments 2 and 3 consisted of *M. ovipneumoniae* (50 and 250 ug, respectively) in 500 ul PBS emulsified in an equal volume of Freund's Incomplete (oil) adjuvant.

### *M. ovipneumoniae* challenge of Experiment 1 animals

Animals from Experiment 1 (N = 4) and naturally exposed domestic sheep (N = 6) were challenged with *M. ovipneumoniae* strain DS11-14153 in order to assess resistance to colonization. The naturally exposed sheep originated from the source flock of strain DS11-14153, the source of the strain whose bacterin was evaluated in the immunization experiments. Colonization inocula consisted of nasal washes from DS11-14153-colonized domestic sheep, pooled and diluted to a final volume of 600 ml in PBS, mixed and then divided into 60 ml challenge aliquots. Inocula were administered into nares, conjunctival sacs and oral cavities of the sheep on day 84 (i.e., 56 days following the second immunization dose of Experiment 1). For this challenge study, nasal swabs for RT-PCR detection of *M. ovipneumoniae* were collected daily on days 83–98 and again on day 128, but test results from the six naturally exposed sheep at numerous additional times prior to and after this study were also available for comparison.

### Serum neutralization of *M. ovipneumoniae*

Since the cELISA assay simply detects binding antibody, a functional assay for *M. ovipneumoniae* neutralizing antibodies was also performed using a metabolic inhibition format described in [31], but modified to use ATP content as a measure of viable bacterial cell volume in place of the color change units described. Briefly, the ATP-biomass kit HS (BioThema AB, Handen, Sweden) was used according to the manufacturer's instructions to quantitate *M. ovipneumoniae* cell density by luminometry (TD 20/20 Luminometer, Turner Designs, Sunnyvale, CA, USA) at 0 and 24 hrs after combining test serum (50 ul) with SP4 broth inoculated (1∶50 v/v) with an overnight culture (37°C, 5% CO_2_) of *M. ovipneumoniae* DS2011-14153. Broth inhibition was determined in triplicate for the adult sheep sera and in duplicate for the lamb sera (due to volume limitations).

### Statistical analysis

cELISA data (%I) and serum inhibition data from immunized and control animals in Experiment 3 were compared by pooled t-test (2-tailed) [32]. The serum inhibition had separate analyses for day 0 serum (pre-immunization) data, day 42 serum (post-immunization) data, and lamb serum (passive immunization) data. *P*<0.05 was considered statistically significant.

## Results

### Experiment 1

This experiment was designed to determine whether live *M. ovipneumoniae* cells administered subcutaneously would induce local infection, significant local inflammatory responses and/or measureable *M. ovipneumoniae* specific antibody responses. All four experimental sheep were negative for *M. ovipneumoniae* antibody as determined by cELISA and for colonization by PCR on day 0. The immunizations induced no significant changes in complete blood count or fibrinogen levels. No local inflammation was noted at the immunization sites by either direct observation or by histologic evaluation of skin biopsies. All sheep remained non-febrile during the 7 days after the initial inoculation. Full thickness skin biopsies of the immunization sites were negative for *M. ovipneumoniae* by enrichment culture and PCR.

No *M. ovipneumoniae* specific antibody responses were detected by cELISA in the two previously unexposed animals, but the two previously infected animals developed cELISA scores indicative of exposure (i.e., %I>50%) by day 28 of the experiment (Table 2).

**Table 2:** *M. ovipneumoniae* specific antibody responses by cELISA and nasal carriage by RT-PCR after immunization of domestic sheep.

	Day 0		Day 14		Day 28		Day 42		Day 56	
Experiment/Group	%I^1^	PCR^2^	%I	PCR	%I	PCR	%I	PCR	%I	PCR
**Experiment 1**										
**Naïve**	−31 (24)	0/2	−26 (20)	0/2	−9 (23)	0/2	−22 (14)	0/2	−13 (5)	0/2
**Previously infected**	−4 (14)	0/2	56 (12)	0/2	51 (8)	0/2	46 (5)	0/2	23 (24)	0/2
**Experiment 2**	39 (8)	4/5	51 (12)	2/5	44 (14)	3/5	46 (24)	1/5	43 (23)	2/4
**Experiment 3:**										
**Immunized adults**	−11 (23)	0/5	58 (25)*	0/5	67(34)**	0/5	86 (8)**	0/5	83 (9)**	0/5
**Immunized lambs**									73 (31)	
**Control adults**	5 (7)	0/5	25 (6)	0/5	7 (11)	0/5	14 (8)	0/5	10 (7)	0/5
**Control lambs**									7 (12)	

1 I% = Mean (SD) percent inhibition score by cELISA. Higher values indicate increased competition by serum antibodies for the epitope targeted by the monoclonal anti-*M. ovipneumoniae* antibody.

2 PCR = N detected with *M. ovipneumoniae* in nasal secretions by PCR/N tested.

*, ** = Immunized group had a significantly higher %I compared to controls, P<0.05 and<0.01 respectively.

### M. ovipneumoniae challenge study

The potential protective effects of the cELISA antibody responses detected in Experiment 1 animals were evaluated by challenge with *M. ovipneumoniae* on day 84 following the first immunization (Table 3). Non-immunized control sheep (N = 6) from the flock of origin of the challenge strain were identically challenged on the same day to serve as a naturally exposed control group. Following challenge, the immunized but previously unexposed Experiment 1 sheep (N = 2) shed the challenge strain in nasal secretions on >90% of 15 sampling occasions over the following 30 days. The Experiment 1 sheep that were immunized following previous exposure and clearance (N = 2) shed the challenge strain in nasal secretions on 50.0% of 15 samples each over the following 30 days, a rate similar to that of the naturally exposed but non-immunized controls (47.6%). The results indicated the possibility of significant individual variation in resistance or immunity to *M. ovipneumoniae* colonization. Specifically, the naturally exposed group included three animals that had been consistently negative for nasal colonization of 5 or more samples prior to challenge; nasal colonization of these three animals was less frequently detected both during the 14 days following challenge and subsequently. In contrast, the other three naturally exposed animals with frequent detection of *M. ovipneumoniae* in nasal secretions prior to challenge (12 of 18 samples, 67%) were similarly more likely to be nasally colonized following challenge (Table 3).

**Table 3:** *M. ovipneumoniae* nasal colonization following challenge of naïve immunized sheep, previously infected immunized sheep, and naturally exposed sheep in Experiment 1.

Group	N	Pre-challenge (days −46 to −752)	Post-challenge (days 1 to 14)	Post-challenge (days 30 to 315)
**Immunized sheep**				
**Naïve**	2	0/14 (0%)^1^	26/28 (92.9%)	2/2 (100%)
**Previously infected**	2	0/14 (0%)	14/28 (50%)	2/2 (100%)
**Naturally exposed sheep**				
**Previous low shedders**	3	0/17 (0%)	9/42 (21.4%)	6/27 (22.2%)
**Previous high shedders**	3	12/18 (66.7%)	31/42 (73.8%)	25/29 (86.2%)

1 PCR = N *M. ovipneumoniae* PCR positive nasal secretions/N tests (%).

### Experiment 2

Experiment 2 utilizing sheep originating in a *M. ovipneumoniae* positive flock, was designed to determine whether the addition of oil adjuvant and the use of sheep originating in a *M. ovipneumoniae* positive flock (previously exposed and immunologically primed to this pathogen) would improve immunogenicity. Prior to inoculation (day 0), one of the five sheep was antibody positive by cELISA, and four of the five sheep carried *M. ovipneumoniae* in the nose as detected by RT-PCR. However, once again no consistent cELISA antibody responses were detected in this experiment (Table 2). At the end of the study, two sheep were antibody positive by cELISA and three carried *M. ovipneumoniae* in the nose as detected by RT-PCR, while two sheep remained both antibody and RT-PCR negative. Evidence of local inflammation (heat, swelling) at the right axillary injection sites were observed during the first week after immunization. No evidence of systemic inflammation was revealed by complete blood counts or fibrinogen tests, and no febrile responses were detected. Well circumscribed 1 cm diameter non-painful plaques were observed at injection sites on days 15 and 43, and histopathology of biopsies obtained on these days revealed lymphocytic inflammation in the right axilla. All biopsy specimens were RT-PCR negative for *M. ovipneumoniae*.

### Experiment 3

Given the lack of detectable *M. ovipneumoniae* antibody responses in experiments 1 and 2, the antigen dose was increased to 250 ug for Experiment 3. All ten sheep were antibody negative by cELISA and all nasal swab samples were RT-PCR negative on day 0, and all animals remained RT-PCR negative on all subsequent samples. Control group sheep remained cELISA negative through the experiment, and their lambs did not acquire detectable passive anti-*M. ovipneumoniae* antibodies. Immunized sheep developed significant cELISA antibody responses and the lambs born to immunized ewes acquired passive anti-*M. ovipneumoniae* antibodies (Table 2).

Adverse reactions to the first immunization dose were limited to local reactions (heat, swelling) at immunization sites. The day following the second immunization dose (day 29), four of the immunization group sheep developed local reactions that progressed to well circumscribed, non-painful plaques by day 35. One sheep developed a transient gait abnormality attributed to injection site soreness. One sheep in the immunized group developed a sterile injection site abscess on day 21 that was treated by drainage and flushing and required re-treatment on day 56. Complete blood counts remained within normal limits on all sheep on day 31 (3 days after booster immunization) but transient fevers (39.8 to 41°C) and elevated fibrinogen values (500–700 mg/dL) were observed.

**Figure 1:**
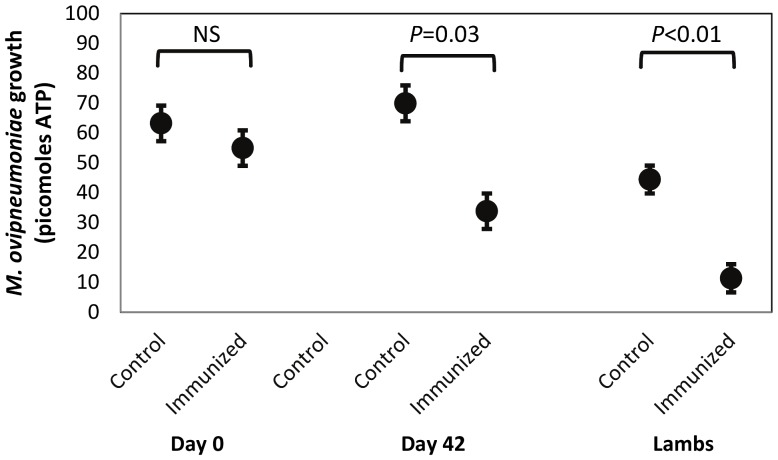
Inhibition of *M. ovipneumoniae* growth was assessed using serum from control (non-immunized) and immunized sheep prior to (day 0) and following (day 42) the immunization protocol, and in serum from lambs born to the experimental ewes. ATP (picomoles) was used as a proxy for *M. ovipneumoniae* cell volume in broth cultures 24 hrs after addition of a standardized inoculum. Significantly less *M. ovipneumoniae* ATP accumulation was observed in the presence of serum from immunized ewes and their lambs.

Serum inhibition of *M. ovipneumoniae* was evident using the broth inhibition test. Serum obtained from immunization group sheep at day 42 limited *M. ovipneumoniae* growth as determined by ATP quantitation compared to day 0 sera or compared to control group sheep (Figure 1). In addition, sera from lambs of immunization group ewes inhibited *M. ovipneumoniae* growth in broth compared to sera from lambs of control group ewes (Figure 1).

## Discussion

*Mycoplasma ovipneumoniae* is a primary infectious agent of epizootic pneumonia in bighorn sheep when introduced into naïve bighorn sheep populations following contact with domestic small ruminants [1], [2], [5], [6], [21]. Immunization of bighorn sheep could potentially protect them from *M. ovipneumoniae* infection, but immunization of wild bighorn sheep would be costly and difficult to impossible to accomplish and maintain. Immunization of domestic small ruminants adjacent to bighorn sheep habitat may represent a more practical and feasible method to reduce the risks of disease transmission. The potential value of reducing *M. ovipneumoniae* shedding by domestic sheep was demonstrated by an experimental contact study between *M. ovipneumoniae*-free domestic sheep and bighorn sheep that did not result in epidemic pneumonia in the bighorn sheep. This contact study is in contrast to previous domestic sheep contact studies in which *M. ovipneumoniae* was not excluded and had resulted in >95% bighorn sheep pneumonia mortality [1], [2], [5], [21].

The present study was designed to explore the safety and immunogenicity of *M. ovipneumoniae* immunization of domestic sheep as the first step in evaluation of immunization to reduce *M. ovipneumoniae* shedding. The results clarify some of the criteria necessary to produce detectable antibody responses to *M. ovipneumoniae* in domestic sheep. For instance, experiments 1 and 2 demonstrated that 50 ug *M. ovipneumoniae* whole cell protein, either as live whole cells or emulsified in oil adjuvant, was insufficient to elicit consistent antibody responses in either naïve and previously exposed sheep. In contrast, increasing the antigen dose to 250 ug for Experiment 3 resulted in strong antibody responses in ewes and passive transfer of antibodies to lambs. It may well be that an intermediate antigenic masses, perhaps measured by a more precise protein measure than the Bradford Assay utilized in the present study, will prove sufficient for inducing antibody responses in domestic sheep.

This study clarified the association between serologic assays that detect antibodies that bind to *M. ovipneumoniae* and those that detect antibodies capable of inhibiting growth of the agent. Previously, indirect hemagglutination and ELISA assays have proven value in epidemiologic studies of associations between pneumonia status and *M. ovipneumoniae* exposure in bighorn and domestic sheep [1], [6], [22], [23], [33], [34]. The results of this study confirmed that cELISA seroconversion was also associated with the development of antibodies capable of inhibiting the growth of *M. ovipneumoniae*.

While the demonstration of growth inhibiting antibodies is promising, it still will be necessary to conduct challenge experiments to determine whether development of these antibodies affects nasal carriage and shedding of *M. ovipneumoniae* in previously colonized animals. In experiments 1 and 2, we attempted to immunize sheep that were previously or currently colonized with *M. ovipneumoniae*, attempting to mimic the conditions present in most real world sheep flocks. The results of the *M. ovipneumoniae* colonization trial clearly demonstrated the lack of protection by the immunization regime of Experiment 1, consistent with the lack of seroconversion of those animals, as well as demonstrating that relatively little resistance to reinfection. Ideally, a challenge study would have also been conducted on the sheep in Experiment 3, but this was not possible because the animals were required for another research program. Therefore, assessment of *in vivo* protection in immunized animals will be needed to assess their ability to reduce *M. ovipneumoniae* nasal colonization and shedding. Similarly, while we demonstrated passive transfer of *M. ovipneumoniae* binding and growth inhibiting antibodies to lambs, the potential protective effects of these antibodies against *M. ovipneumoniae* will need to be determined in future studies.

There is precedent for control of *Mycoplasma* spp. infections in other domestic animals through immunization. However, it has not been possible to date to develop efficacious broadly protective vaccines to the closely related *M. hyopneumoniae*, the etiologic agent of porcine enzootic pneumonia [27], [35], [36]. *M. hypopneumoniae* shares the same basic pathophysiology and broad strain diversity as *M. ovipneumoniae* [2], [23], [27]. The demonstration of only partial protection from disease and little if any effect on *M. hyopneumoniae* colonization, illustrates the challenge of vaccination development for some respiratory mycoplasmal diseases and may give insight into the challenges faced in the future development of an effective *M. ovipneumoniae* vaccine.

Effective immunization of sheep with *M. ovipneumoniae* could assist with prevention of respiratory disease in domestic sheep and may also reduce the risk of transmission of *M. ovipneumoniae* to bighorn sheep. This study has produced information on safety and immunogenicity that are the first steps in the development of immunization strategies targeting *M. ovipneumoniae* infections of domestic sheep. We demonstrated the need for relatively large (>50 ug) antigenic mass to produce detectable serum antibody responses, the parallel development of blocking antibodies detected by cELISA and growth inhibiting antibodies, the potential for significant adverse local (heat, swelling) and systemic (fever, elevated fibrinogen levels) effects of immunization, and the ability of *M. ovipneumoniae* specific antibodies to be passively transferred to lambs. Future studies will be required to define the ability of subcutaneous immunization to elicit mucosal antibodies, which are presumably required to impact *M. ovipneumoniae* infection of the nasal cavity.
